# Incidence of Online Health Information Search: A Useful Proxy for Public Health Risk Perception

**DOI:** 10.2196/jmir.2401

**Published:** 2013-06-17

**Authors:** Bo Liang, Debra L Scammon

**Affiliations:** ^1^Department of MarketingDavid Eccles School of BusinessUniversity of UtahSalt Lake City, UTUnited States

**Keywords:** health risk perception, social influence, ecological system

## Abstract

**Background:**

Internet users use search engines to look for information online, including health information. Researchers in medical informatics have found a high correlation of the occurrence of certain search queries and the incidence of certain diseases. Consumers’ search for information about diseases is related to current health status with regard to a disease and to the social environments that shape the public’s attitudes and behaviors.

**Objective:**

This study aimed to investigate the extent to which public health risk perception as demonstrated by online information searches related to a health risk can be explained by the incidence of the health risk and social components of a specific population’s environment. Using an ecological perspective, we suggest that a population’s general concern for a health risk is formed by the incidence of the risk and social (eg, media attention) factors related with the risk.

**Methods:**

We constructed a dataset that included state-level data from 32 states on the incidence of the flu; a number of social factors, such as media attention to the flu; private resources, such as education and health insurance coverage; public resources, such as hospital beds and primary physicians; and utilization of these resources, including inpatient days and outpatient visits. We then explored whether online information searches about the flu (seasonal and pandemic flu) can be predicted using these variables. We used factor analysis to construct indexes for sets of social factors (private resources, public resources). We then applied panel data multiple regression analysis to exploit both time-series and cross-sectional variation in the data over a 7-year period.

**Results:**

Overall, the results provide evidence that the main effects of independent variables—the incidence of the flu (*P*<.001); social factors, including media attention (*P*<.001); private resources, including life quality (*P*<.001) and health lifestyles (*P*=.009); and public resources, such as hospital care utilization (*P*=.008) and public health funds (*P*=.02)—have significant effects on Web searches for queries related to the flu. After controlling for the number of reported disease cases and Internet access rate by state, we estimate the contribution of social factors to the public health risk perception levels by state (*R^2^*=23.37%). The interaction effects between flu incidence and social factors for our search terms did not add to the explanatory power of our regression models (*R^2^*<1%).

**Conclusions:**

Our study suggests a practical way to measure the public’s health risk perception for certain diseases using online information search volume by state. The social environment influences public risk perception regardless of disease incidence. Thus, monitoring the social variables can be very helpful in being ready to respond to the public’s behavior in dealing with public health threats.

## Introduction

The Internet has rapidly become an important source of health information: 61% of American Internet users have searched for health information online [1]. Most American Internet users primarily use search engines to look for information including health information [2-4]. Researchers in medical informatics have found a high correlation of the occurrence of certain search queries and the incidence of certain diseases, especially infectious diseases (eg, the flu), and thus have suggested the use of search query data for syndromic surveillance, or early detection of outbreaks [5-10]; this body of research has been well framed [11-16] and termed *infodemiology* by Eysenbach [6,17].

The existence of a correlation between search query volume and disease outbreaks raises a number of questions: Is the occurrence of certain search queries fully accounted for by the incidence of certain diseases? Do consumers search for online information related to a certain disease only when they have symptoms related to the disease? Are there situations in which consumers without any symptoms related to the disease search for online information related to the disease?

The perception of risk can play a role in many consumer decisions. Risk perception is the judgment that people make about the characteristics and severity of risks [18]. Over the past few decades, considerable research has been conducted on risk perception. The traditional theories of risk perception (eg, expected utility theory, prospect theory) were established by work in behavioral economics that focused on individuals’ statistical or heuristic estimation of the value of alternative choices [19-22]. Understanding the risks perceived by individuals, and collectively by populations, is very helpful as the basis for designing effective strategies for communicating about risks. As a result, risk perception and risk communication have been used extensively in the field of public health [23,24]

Ecological systems theory holds that people interact with multiple social systems (eg, cultures, communities) in an environment [25,26]. Since its first introduction, ecological systems theory has been applied in various areas, such as health promotion [27]. From the ecological systems perspective, members of a specific population are influenced by the same sociocultural factors. Thus, their collective behavior is shaped by common factors.

We suggest that online information searches related to a health risk reflect the public’s collective perception for the risk, which is associated not only with current health status (eg, the incidence of a disease), but also with the social environments related to the risk (eg, availability of public health resources) [28]. Our study extends previous work by exploring the association between online health information search and multiple sociocultural factors related to public health risk perception.

We selected online information searches related to the flu as the object of this study. The seasonal flu occurs on a regular basis; in the United States, an average of 5% to 20% of the population gets seasonal flu and more than 200,000 people are hospitalized annually from seasonal flu-related complications. Thus, a significant proportion of the population has direct experience with the flu. Further, the flu can cause mild to severe illness and consumers are generally aware of the risks related to flu. In this study, we demonstrate the extent to which online information searches for the flu is explained by the incidence of the flu (including seasonal flu and pandemic flu) and sociocultural components of a population’s environment. We suggest that the occurrence of online information search related to a health risk can be a practical way to assess the public’s general concern for the risk, or public health risk perception.

### The Ecological View of Risk Perception

From an ecological systems point of view, individuals grow and develop in different layered environmental systems, such as family, school, neighborhood, and community [25]. Because risk perception is a sociocultural construct [29,30], individuals form their perception of risks under the influence of the sociocultural components within these systems. Individuals form their risk perception through 2 types of experiences: direct (personal) and indirect (social) experience with the risk [30,31]. For a specific risk, some members of the population may directly experience the risk (eg, patients during a pandemic and victims of a natural disaster) whereas others may experience the risk only vicariously (eg, through exposure to media accounts of the event).

Both groups have social experience with the risk through sociocultural activities, such as receiving information about the risk from multiple social sources (eg, news media, personal social networks), and interpreting the information based on certain values or cultural biases [29-31]. Thus, individuals in a particular ecological system form their perceptions about a risk in response to a set of sociocultural components in the environment (eg, news coverage by mass media, demographics). Because these individuals share the same sociocultural environment, their risk perceptions have features in common. Individuals’ risk perceptions are formed on the basis of a constellation of direct and indirect experience with a risk (sociocultural factors), and the interaction of the 2 types of experiences. The dynamic socioculturalization process through which individuals’ risk perceptions evolve over time leads to the formation of population risk perception.

As an example, individuals who live in a specific community may form their perception about the risk of smoking influenced by their own experience with smoking and a set of shared sociocultural factors, such as the news coverage by the local and national media about the risks of smoking and the behavioral norms of other individuals in their community. As this dynamic process continues over time for individuals within a community, public risk perception for smoking will develop.

Building from this, our main proposition is that public risk perception is predicted by individuals’ direct (personal) and indirect (social) experiences with a risk, and the interaction of the 2 experiences over time within an ecological system.

Previous research has identified a strong correlation between online information search and the incidence of diseases [5-10]. In the following, we establish our hypotheses related to the social experiences that shape public risk perception. Building from previous literature on risk perception, especially health risk perception, we identify 2 major categories of social factors that are associated with public health risk perception: (1) news coverage by mass media, and (2) the availability of resources (including private and public resources).

### Media Coverage

Agenda-setting theory proposes that mass media have an important influence on what issues the public considers to be important [32]. A number of studies have found powerful effects of mass media on individual risk perception [33-36]. For example, one study found that the number of news articles about the H1N1 pandemic was positively associated with individual preventive pharmaceutical intervention and engagement in information seeking [36]. It is important to note that media coverage may not always be factually correct. When the coverage of health risks by mass media is misleading (eg, exaggeration or stigmatization), the public may form misperceptions of the characteristics of the risks [37,38]. Regardless of the content of media coverage, the extent of coverage likely affects public risk perception.

We argue that when mass media pay more attention to a health risk by increasing coverage of the risk, the public will have higher awareness of the risk. Further, we suggest that when there is a high incidence of a health risk, the public will become more sensitive to the attention paid by media reporting. Their concern for the risk will be higher as the media attention increases. Thus, our first hypotheses are:

H1a: Online information search related to a health risk will be higher when mass media attention to the risk is higher;

H1b: The effect of the incidence of a health risk on online information search related to the risk will be greater when mass media attention to the risk is greater.

### Availability of Resources

Research has shown that the availability of resources can reduce an individual’s perceived risk [39-42]. We classify the resources related to health risk perception into 2 categories: private resources and public resources. Private resources are the resources that individuals can acquire through their own efforts, such as financial, informational, physiological, and physical resources. Studies have found that the availability of private resources is negatively associated with health risk perceptions. Three types of private resources are particularly important in the context of health risk perceptions: life quality (eg, education [43], family income [44]), health status [45,46], and health lifestyles (eg, tobacco and alcohol consumption [47]).

Public resources are the resources that the public obtains from organizations such as charities and government. Little research has been conducted on the association between public resource support and risk perception. We classify public resources into 4 groups: natural resources (eg, population density, especially risks that are related to natural disasters), financial resources (eg, funding for public health), capacity of public resources (eg, hospital beds), and utilization of public resources (eg, hospital admissions). In our study, we use capacity and utilization of public health resources as measures of availability of health care resources. Because natural and financial resources and capacity and utilization of public resources are important resources with which the public can deal with health risks, we assume that the availability of these public resources is negatively associated with public health risk perception. Further, for those experiencing a health risk the availability of public resources may be particularly critical. Thus, we expect that their risk perception will be more likely to be influenced by the availability of resources.

We propose our second hypotheses:

H2a: Online information search for a health risk will be lower when the availability of private resources represented by life quality, health status, and health lifestyles, and public resources represented by natural and financial resources and capacity and utilization of public services is greater;

H2b: The effect of the incidence of a health risk on online information search related to the risk will be greater when the availability of private and public resources is lower.

## Methods

Our study aims to explore the relationship between online information searches related to the flu and factors related to public health risk perception for the flu, including the incidence of the flu and the social factors related to the flu (news coverage and availability of resources). We used data from 2004 to 2011 from multiple published sources as detailed in the following section. The unit of analysis of our study is state population. In the following sections, we first detail the measures and data collection process for each variable necessary to test our hypotheses, and then present our analysis.

### Measures

#### Online Information Search

Following methods used in previous studies [47-49], in this study we use Google Insights for Search (GIFS) to identify the changing patterns of Web searches used by consumers for queries related to the flu. Details of GIFS methodology are presented in Multimedia Appendix 1.

Research has shown that Internet users usually include 1 or 2 terms in a search query [50]. Thus, for each of our search queries we include 1 or 2 related terms. People may have specific concerns related to prevention, diagnosis, and treatment of the flu. Thus, we preselected 96 search queries based on 3 categories: prevention (eg, flu shots, flu prevention), diagnosis (eg, flu symptoms, flu fever), and treatment of the flu (eg, Tamiflu). Our criterion for query selection was the availability of weekly search volume data for queries for 25 states in the United States (if there is not enough search volume for each query by state, GIFS shows only monthly search volume or no results). After checking the search volumes for these preselected queries, we identified 2 queries that fulfilled our criterion: flu shot(s) and flu symptom(s). The prevalence of the 2 search queries shows that the most common response by the public to the flu is to take preventive actions and to determine whether they have contracted the flu. In our study, we use the search volumes for flu shot(s) and flu symptom(s) to represent the state population’s risk perception for prevention and diagnosis of the flu, respectively.

#### Flu Incidence

Public health agencies in the United States often track the percentage of outpatient visits related to influenza-like illness (ILI), collected through the US Influenza Sentinel Provider Surveillance Network [51]. A high ILI percentage indicates that a large fraction of patients are experiencing flu-like symptoms. Based on previous studies of the correlation of Web search and flu surveillance [9,52] and the availability of data, we used weekly ILI outpatient visit rates to measure the weekly incidence of the flu by state (like search data, this measure is automatically normed for the state’s population). We gathered these data from the official website of the Department of Health for each state. The data are not available for all states and all observed years. In all, the dataset includes the weekly ILI rate data for between 15 and 31 states over the time period of 2004 to 2011.

#### Mass Media Attention

Previous research on the influence of mass media on risk perception has used the number of news articles to measure mass media attention at the national level [36]. Because states vary in their population, we use the number of news articles per 1 million population to measure the relative media attention for state populations. We collected weekly data on the number of news articles by using the news search function in LexisNexis Academic [53], a comprehensive database of national and regional news media. To find news articles that focused on the topic *flu*, we set the search term in GIFS as flu and the restriction as “headline & lead.” We set the time intervals as those used for search volume data from GIFS, and the sources of news as US newspapers and wires. We also set the article location (articles about a geographic location) as each state, indicating that the articles cover the population of a specific state. We collected annual state population data from the website of the [54]. The data for media attention covers all the states and all the observed years.

#### Private and Public Resources

For private resources, we have variables indicating health status, life quality, and health lifestyles. According to the Centers for Disease Control and Prevention (CDC), the population groups most vulnerable to the flu are young children under age 5 years, the population aged over 65 years, pregnant women, and the population with chronic diseases, such as HIV [55]. Based on the availability of data, we included 2 variables indicating age-related health status: the percentages of the population under 5 years and over 65 years, and 2 variables indicating chronic disease-related health status: the percentages of the population that have asthma and diabetes. We also included variables indicating life quality: the percentage of the population that has completed a bachelor’s degree, median household income, the percentage of the population that reported good health status, and the health insurance coverage rate. For health lifestyles, we included variables indicating the percentage of the population that used tobacco, exercised regularly, and was overweight or obese. Preventive health behavior is an important part of health lifestyles. Thus, we included a variable indicating the percentage of people over 65 years old who have had flu shots.

For public resources, we included variables indicating natural and financial resources, and capacity and utilization of public resources. Because the flu is a contagious respiratory disease, we used population density as a measure of natural resources. The flu is a health-related risk; therefore, we used public health funding as a measure of financial resources. We use the number of primary physicians per 1 million population as a measure of capacity of ambulatory care, outpatient visits per 1000 population as a measure of utilization of ambulatory care, the number of hospital beds per 1000 population as a measure of hospital care capacity, and hospital admissions, emergency room visits per 1000 population, and inpatient days as a measures of utilization of hospital care. We collected the data for private and public resources from the websites of the US Census Bureau [56], the CDC [57], Kaiser State Health Facts [58], and Trust for America’s Health [59]. A majority of the data about health status is captured through state residents’ self-report surveys conducted by the CDC. In all, our dataset for private and public sources includes 20 annual variables. The data for each variable are available for all states (Hawaii is an exception because the data were lacking for the years 2004 and 2005) and for at least 4 years for the observed time period. We present the details about our measures in Tables 1 and 2.

### Analysis

To include as many observations and variables as possible, we use unbalanced panel data in our analyses. According to the CDC, the official annual flu season starts in October and ends in May covering 33 weeks [60]. As most of the states in our dataset have missing values for the incidence of the flu in some weeks outside of each flu season (especially in the years before the H1N1 flu pandemic occurred), we dropped the observations for these weeks. Because the Web search volume data has been normalized by the total Internet traffic from each respective state, we included control variables representing household Internet usage, the percentage of households with an Internet connection, and the percentage of households with an Internet connection through broadband for each state. These data were obtained from the website of the US Department of Commerce and were available for all of the states for 3 years: 2007, 2009, and 2010 [61].

**Figure 1:**
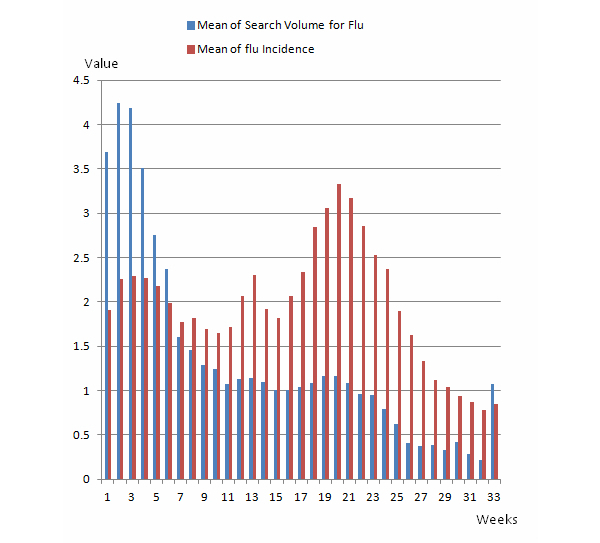
Trends of the means of search volumes for flu and the incidence of the flu.

To account for weekly variations in search volumes, we included 33 dummy variables to indicate the specific weeks in each flu season. We also observed that in the 2 flu seasons following the 2009 H1N1 flu pandemic, search volumes for flu-related queries were higher than in the flu seasons before the pandemic occurred. To account for this variation, we used a dummy variable to indicate the weeks before and after the H1N1 flu pandemic. We present the trends of the means of the search volumes for flu and the incidence of the flu for all the states across the 33 weeks in each flu season in Figure 1.

**Table 1:** Study variables: measures and types of data.

Variable			Measure	Type of data
Search for symptoms			Web search volume for flu symptom(s)	Nonofficial public databases
Search for shots			Web search volumes for flu shot (s)	Nonofficial public databases
Flu incidence			Influenza-like illness (ILI) outpatient visits	Official reports
Mass media attention			The number of news articles per 1 million population	Nonofficial public databases
**Private resources**				
	**Age-related health status**			
		Population <5 years	The percent of population <5 years	Official reports
		Population >65 years	The percent of population >65 years	Official reports
	**Chronic disease-related health status**			
		Asthma	Percent of adults who have been diagnosed with asthma	Self-report surveys
		Diabetes	Percent of adults who have been diagnosed with diabetes	Self-report surveys
	**Life quality**			
		Bachelor’s degree	Percent of population that has completed a bachelor’s degree	Official reports
		Income	Median household income	Official reports
		Good health	Percent of population that has fair or better health	Self-report surveys
		Health insurance coverage	Percent of adults aged 18-64 who have any kind of health care coverage	Self-report surveys
		Exercises	Percent of adults who indicated that they participated in physical activities during the past month	Self-report surveys
	**Health lifestyle**			
		Smoking	Percent of adults who are currently smokers	Self-report surveys
		Overweight or obese	Percent of adults who are overweight or obese	Self-report surveys
		Flu shots	Percent of adults ≥65 who have had a flu shot within the past year	Self-report surveys
**Public resources**				
	**Natural resources**			
		Population density	Population per square mile (land area)	Official reports
	**Financial resources**			
		Public funds	Total of public health funding per capita^a^	Official reports
	**Ambulatory care capacity**			
		Primary physicians	Number of primary care physicians per 1 million population^b^	Nonofficial public databases
	**Ambulatory care utilization**			
		Outpatient visits	Number of outpatient visits per 1000 population^c^	Nonofficial public databases
	**Hospital care capacity**			
		Hospital beds	Number of beds per 1000 population	Nonofficial public database
	**Hospital care utilization**			
		Hospital admissions	Number of hospital admissions per 1000 population^c^	Nonofficial public databases
		Emergency department visits	Number of emergency department visits per 1000 population^c^	Nonofficial public databases
		Inpatient days	Number of inpatient days per 1000 population^c^	Nonofficial public databases

^a^Data includes funds from CDC and State health agencies.

^b^Data includes physicians for general practice, family practice, OB-GYN, pediatrics, and internal medicine.

^c^Data are for community hospitals, which represent 85% of all hospitals. Federal hospitals, long-term care hospitals, psychiatric hospitals, institutions for persons with mental disabilities, and hospitals for alcoholism and other chemical dependencies are not included.

**Table 2:** Study variables: data availability and sources.

Variable			Frequency data were collected	Number of years data are available	Data sources
Search for symptoms			Weekly	7	Google Insights for Search
Search for shots			Weekly	7	Google Insights for Search
Flu incidence			Weekly	7	Public health agencies
Media			Weekly	7	LexisNexis Academic
**Private resources**					
	**Age-related health status**				
		Population <5 years	Annually	7	US Census Bureau
		Population >65 years	Annually	7	US Census Bureau
	**Chronic disease-related health status**				
		Asthma	Annually	7	CDC
		Diabetes	Annually	7	CDC
	**Life quality**				
		Bachelor’s degree	Annually	5	US Census Bureau
		Income	Annually	7	US Census Bureau
		Good health	Annually	7	CDC
		Health insurance coverage	Annually	7	CDC
		Exercises	Annually	7	CDC
	**Health lifestyle**				
		Smoking	Annually	7	CDC
		Overweight or obese	Annually	7	CDC
		Flu shots	Annually	7	CDC
**Public resources**					
	**Natural resources**				
		Population density	Annually	7	US Census Bureau
	**Financial resources**				
		Public funds	Annually	5	Trust for America’s Health
	**Ambulatory care capacity**				
		Primary physicians	Annually	6	Henry J Kaiser Family Foundation
	**Ambulatory care utilization**				
		Outpatient visits	Annually	6	Henry J Kaiser Family Foundation
	**Hospital care capacity**				
		Hospital beds	Annually	6	Henry J Kaiser Family Foundation
	**Hospital care utilization**				
		Hospital admissions	Annually	6	Henry J Kaiser Family Foundation
		Emergency department visits	Annually	6	Henry J Kaiser Family Foundation
		Inpatient days	Annually	6	Henry J Kaiser Family Foundation

#### Data Transformation

We normalized the data for all continuous variables included in the models by natural log transformations. To make the coefficients for interaction effects more interpretable, we centered all the continuous independent variables by subtracting the mean from each value.

#### Factor Analysis

Table 3 presents summary statistics for all dependent and independent variables included in the study. We used Stata (StataCorp LP, College Station, TX, USA) to perform factor analysis with varimax rotation to reduce the number of independent variables. Because factor analysis by Stata is conducted on the correlations (as opposed to the covariances), it is not a concern that the variables have different means and/or standard deviations (eg, variables are measured in different scales). Based on the components identified by factor analysis, 5 composite indexes were constructed: (1) life quality index, (2) age-related health status index, (3) chronic disease-related health status index, (4) health lifestyle index, and (5) hospital care utilization index. We used the values of the composite indexes generated by factor analysis in our regression models. The life quality index includes positive factors indicating the percentage of the population with a bachelor’s degree, good health status, health insurance, and median household income. The age-related health status index includes a positive factor indicating the percentage of the population younger than 5 years and a negative factor indicating the percentage of the population older than 65 years. The chronic disease–related health status index includes positive factors indicating the percentage of the population that has been diagnosed with asthma and diabetes. The health lifestyle index includes positive factors indicating the percentage of the population that consumes tobacco and are overweight or obese, and negative factors indicating the percentage of the population that exercises regularly and the percentage of the population older than 65 years who have had a flu shot. The hospital care utilization index includes positive factors indicating the number of hospital admissions, emergency department visits, and inpatient days. All other variables are represented by single data items. We present factor loadings and the uniqueness for each variable in Table 4.

#### Model Construction

We applied panel data multiple regression analysis to exploit both time-series and cross-sectional variation in the data using Stata. We built regression models to examine the effects of the incidence of flu, social factors including media attention, and private and public resources and their interaction on the Web search volumes for the 2 queries: flu symptom(s) and flu shot(s). Because we assumed that variation across states was random and uncorrelated with the independent variables, we used random state-specific effects in our models. We also used robust Huber–White standard errors to address any potential heteroscedasticity and autocorrelation in our estimation. To avoid collinearity, we examined the correlation matrix of independent variables (Table 5). We found that each pair of variables has a correlation coefficient of less than 0.8 with most less than 0.6.

To investigate the separate effects of control variables and independent variables on dependent variables, we ran 6 models for each dependent variable (each search query) sequentially as shown in Table 6.

### Results

Overall, the results provide substantial evidence that the main effects of the independent variables we analyzed—the incidence of the flu, media attention, and private and public resources—have significant affects on Web search for queries related to the flu. Specifically, the results provide full support for hypotheses H1a and H1b, and partial support for hypotheses H2a and H2b.

### Control Variables

The models including control variables (Models 1) were significant (*P*<.001) with coefficient of determination (*R* ^*2*^) value of 37.88% and 59.11% for flu symptoms and flu shots, respectively. Specifically, the dummy variables for the occurrence of H1N1 and seasonality were significant in the models for both search queries. However, with the independent variables sequentially added in Models 2 to Models 6, H1N1 occurrence showed significant effects only in the model for flu symptoms, but not flu shots. Seasonality contributed significantly to the variance of Web search for flu shots. We present the results for the control variables in Tables 7 and 8.

#### Flu Incidence and Media Attention

With flu incidence as an independent variable added in model 2, the *r* ^*2*^ values increased slightly by approximately 2% from model 1 for flu symptoms (*P*<.001) and flu shots (*P*<.001). Further, with media attention as an independent variable added in model 3, *r* ^*2*^ values increased approximately 7% and 1% from model 2 for flu symptoms (*P*<.001) and (*P*<.001) flu shots, respectively. With the interaction of flu incidence and media attention added in model 4, the *R* ^*2*^ values showed an increase of less than 1% from model 3 for flu symptoms (*P*<.001) and flu shots (*P*=.003).

All these models were significant (*P*<.001) with flu incidence (*P*<.001), media attention (*P*<.001), and their interaction (*P*<.001 for flu symptoms; *P*=.001 for flu shots) showing positive effects on Web search volume. The changes in *R* ^*2*^ values from model 3 to model 4 show that media attention has a stronger positive influence on a population’s search for flu symptoms than for flu shots.

#### Private and Public Resources

With variables indicating private and public resources added in models 5, the *r* ^*2*^ values showed a substantial increase from model 4 of 23.37% for flu symptoms (*P*<.001) and of 6.28% for flu shots (*P*<.001). For private resources, the life quality index (*P*=.001), health lifestyle index (*P*=.009), and chronic disease index (*P*=.004) had negative effects on search volume for flu symptoms.

For public resources, the number of outpatient visits (*P*<.001) and hospital care utilization index (*P*=.008) had positive effects, and the number of hospital beds had negative effects (*P*<.001) on search volume for flu symptoms. Public health funds (*P*=.02) had a negative effect, whereas population density (*P*=.001) and number of primary physicians (*P*=.006) had positive effects on search volume for flu shots.

With the interaction of these social factors and flu incidence added in models 6, the *R* ^*2*^ values increased slightly from model 5, 1.2% for flu symptoms (*P*<.001), and 0.55% for flu shots (*P*=.004). The interaction of flu incidence and number of primary physicians had a negative effect on search volume for flu symptoms. The interaction of flu incidence and life quality index had a negative effect on search volume for flu shots. We present the results for the changes in the *R* ^*2*^ values and the coefficient results for the independent variables in Tables 9 and 10.

**Table 3:** Summary statistics.

Variable		Observations (n)	Mean	SD	Min	Max
**Dependent variables**						
	Search for symptoms	5364	2.89	6.14	0.00	70.00
	Search for shots	8217	4.29	9.12	0.00	100.00
**Independent variables**						
	Flu incidence	5226	2.04	2.10	0.00	20.43
	Media	8364	0.95	1.69	0.00	31.12
**Life quality**						
	Bachelor’s degree	6006	27.98	4.35	18.70	38.20
	Income	8217	49788.35	7725.76	32875.00	68059.00
	Health insurance coverage	8184	85.39	4.42	71.50	95.70
	Good health	8184	84.78	4.73	35.80	90.00
**Age-related health status**						
	Population <5 years	8217	6.65	0.78	5.23	9.69
	Population >65 years	8217	12.82	1.57	8.61	17.34
**Chronic disease–related health status**						
	Asthma	8151	8.454	1.167	5.900	11.100
	Diabetes	8184	3.96	0.93	0.00	4.62
**Health lifestyle**						
	Exercise	8184	76.59	3.85	66.60	85.80
	Smoking	8184	18.77	3.17	9.10	26.10
	Overweight and obesity	8184	36.43	1.42	25.60	40.70
	Flu shots	8151	69.99	4.29	55.60	80.00
**Natural resources**						
	Population density	8184	224.88	293.28	5.24	1185.32
**Financial resources**						
	Public funds	6006	56.81	31.91	18.39	197.76
**Ambulatory care capacity**						
	Primary physicians	7128	120.69	27.07	78.50	191.30
**Ambulatory care utilization**						
	Outpatient visits	7128	2134.60	676.25	942.00	4370.00
**Hospital care capacity**						
	Hospital beds	7128	2.76	0.83	1.70	5.60
**Hospital care utilization**						
	Hospital admissions	7128	114.49	17.37	81.00	154.00
	Emergency department visits	7128	401.94	72.33	258.00	601.00
	Inpatient days	7128	652.81	173.13	360.00	1210.00

**Table 4:** Composite indexes from factor analysis.

Index and variable		Loading	Uniqueness^a^
**Life quality**			
	Bachelor’s degree	0.89	0.21
	Good health	0.54	0.71
	Income	0.91	0.17
	Health insurance coverage	0.79	0.37
**Age-related health status**			
	Population <5 years	–0.93	0.13
	Population >65 years	0.93	0.13
**Chronic disease-related health status**			
	Asthma	0.75	0.44
	Diabetes	0.75	0.44
**Health lifestyle**			
	Exercise	–0.81	0.34
	Smoking	0.71	0.49
	Overweight and obesity	0.74	0.45
	Flu shots	–0.52	0.73
**Hospital care utilization**			
	Hospital admissions	0.93	0.13
	ED visits	0.75	0.44
	Inpatient days	0.87	0.24

^a^Uniqueness is defined as 1=communality, or the portion not explained by common factor analysis.

**Table 5:** Correlation matrix of independent variables.

Independent variable		1	2	3	4	5	6	7	8	9	10	11	12
1	Flu incidence	1.00											
2	Media	0.16	1.00										
3	Public funds	0.03	0.11	1.00									
4	Population density	–0.02	–0.04	0.05	1.00								
5	Life quality	–0.23	0.09	0.24	0.41	1.00							
6	Age-related health status	–0.18	–0.02	0.03	0.33	0.21	1.00						
7	Chronic disease–related health status	–0.21	0.03	0.12	0.14	0.35	0.33	1.00					
8	Health lifestyle	0.21	–0.10	–0.21	0.01	–0.62	0.19	–0.28	1.00				
9	Primary physicians	–0.13	0.03	0.27	0.64	0.70	0.48	0.43	–0.30	1.00			
10	Outpatient visits	–0.24	0.07	–0.01	–0.06	0.38	0.44	0.43	–0.05	0.30	1.00		
11	Hospital beds	0.07	0.03	0.00	–0.33	–0.19	0.30	–0.26	0.53	–0.17	0.30	1.00	
12	Hospital care utilization	–0.02	–0.02	–0.09	0.17	–0.04	0.57	–0.07	0.60	0.21	0.40	0.80	1.00

**Table 6:** Model construction.

Model	Control variables	Flu incidence	Media	Flu incidence × media	Other social factors	Flu incidence × other social factors
1	Included					
2	Included	Included				
3	Included	Included	Included			
4	Included	Included	Included	Included		
5	Included	Included	Included	Included	Included	
6	Included	Included	Included	Included	Included	Included

**Table 7:** Coefficients for control variables for flu symptom(s).

Control variable		Flu symptom(s)					
		Model 1	Model 2	Model 3	Model 4	Model 5	Model 6
H1N1 mark		1.666^a^	1.956^a^	1.590^a^	1.483^a^	1.027^a^	0.823^a^
Internet connection		–5.397^a^	1.779	3.69	5.237	0.918	1.339
Broadband connection		–0.024	–5.050^a^	–4.516^a^	–4.748^a^	–0.586	–0.105
**Flu season week**							
	1	–0.980^a^	–0.819^a^	–0.710^a^	–0.847^a^	–0.536^a^	–0.568^a^
	2	–0.843^a^	–0.756^a^	–0.608^a^	–0.771^a^	–0.396^a^	–0.454^a^
	3	–0.790^a^	–0.684^a^	–0.562^a^	–0.744^a^	–0.439^a^	–0.477^a^
	4	–0.877^a^	–0.829^a^	–0.608^a^	–0.741^a^	–0.422^a^	–0.439^a^
	5	–0.875^a^	–0.803^a^	–0.565^a^	–0.640^a^	–0.351^a^	–0.348^a^
	6	–0.845^a^	–0.792^a^	–0.538^a^	–0.602^a^	–0.343^a^	–0.339^a^
	7	–1.036^a^	–0.904^a^	–0.634^a^	–0.655^a^	–0.445^a^	–0.431^a^
	8	–1.010^a^	–0.859^a^	–0.631^a^	–0.625^a^	–0.405^a^	–0.400^a^
	9	–1.057^a^	–0.849^a^	–0.680^a^	–0.643^a^	–0.523^a^	–0.504^a^
	10	–1.123^a^	–0.883^a^	–0.667^a^	–0.648^a^	–0.559^a^	–0.527^a^
	11	–1.099^a^	–0.874^a^	–0.676^a^	–0.595^a^	–0.630^a^	–0.580^a^
	12	–0.992^a^	–0.897^a^	–0.568^a^	–0.547^a^	–0.371^a^	–0.345^a^
	13	–1.031^a^	–1.014^a^	–0.678^a^	–0.630^a^	–0.515^a^	–0.492^a^
	14	–1.203^a^	–1.098^a^	–0.823^a^	–0.769^a^	–0.646^a^	–0.592^a^
	15	–1.187^a^	–1.078^a^	–0.742^a^	–0.703^a^	–0.580^a^	–0.546^a^
	16	–1.061^a^	–1.042^a^	–0.703^a^	–0.670^a^	–0.558^a^	–0.519^a^
	17	–0.798^a^	–1.112^a^	–0.730^a^	–0.631^a^	–0.505^a^	–0.480^a^
	18	–0.606^a^	–1.056^a^	–0.713^a^	–0.657^a^	–0.496^a^	–0.462^a^
	19	–0.660^a^	–1.184^a^	–0.755^a^	–0.642^a^	–0.421^a^	–0.371^a^
	20	–0.662^a^	–1.191^a^	–0.799^a^	–0.705^a^	–0.461^a^	–0.416^a^
	21	–0.835^a^	–1.317^a^	–0.964^a^	–0.910^a^	–0.598^a^	–0.559^a^
	22	–1.071	–1.395^a^	–0.931^a^	–0.856^a^	–0.578^a^	–0.549^a^
	23	–1.334^a^	–1.533^a^	–1.078^a^	–1.000^a^	–0.837^a^	–0.807^a^
	24	–1.454^a^	–1.601^a^	–1.171^a^	–1.121^a^	–0.902^a^	–0.889^a^
	25	–1.618^a^	–1.582^a^	–1.246^a^	–1.220^a^	–1.196^a^	–1.169^a^
	26	–1.852^a^	–1.748^a^	–1.306^a^	–1.284^a^	–1.274^a^	–1.245^a^
	27	–2.063^a^	–1.804^a^	–1.307^a^	–1.354^a^	–1.302^a^	–1.292^a^
	28	–2.199^a^	–1.873^a^	–1.450^a^	–1.509^a^	–1.506^a^	–1.487^a^
	29	–2.372^a^	–1.912^a^	–1.510^a^	–1.600^a^	–1.579^a^	–1.576^a^
	30	–2.499^a^	–2.034^a^	–1.649^a^	–1.735^a^	–1.815^a^	–1.790^a^
	31	–2.531^a^	–1.986^a^	–1.534^a^	–1.693^a^	–1.729^a^	–1.762^a^
	32	–2.658^a^	–2.117^a^	–1.694^a^	–1.880^a^	–2.016^a^	–2.045^a^
	33	–2.908^a^	–2.225^a^	–1.761^a^	–1.992^a^	–2.071^a^	–2.094^a^

^a^ *P*<.05.

**Table 8:** Coefficients for control variables for flu shot(s).

Control variable		Flu shot(s)					
		Model 1	Model 2	Model 3	Model 4	Model 5	Model 6
H1N1 mark		0.606^a^	0.493	0.319	0.286	0.091	0.018
Internet connection		–0.887	–1.419	–0.745	–0.376	3.958	0.806
Broadband connection		0.312	0.478	0.832	0.821	–0.049	1.338
**Flu season week**							
	1	2.459^a^	2.254^a^	2.292^a^	2.237^a^	2.297^a^	2.231^a^
	2	2.728^a^	2.355^a^	2.416^a^	2.333^a^	2.469^a^	2.422^a^
	3	2.612^a^	2.212^a^	2.265^a^	2.163^a^	2.359^a^	2.308^a^
	4	2.342^a^	1.896^a^	2.000^a^	1.925^a^	2.101^a^	2.060^a^
	5	2.244^a^	1.887^a^	2.008^a^	1.966^a^	2.222^a^	2.193^a^
	6	1.811^a^	1.525^a^	1.668^a^	1.631^a^	2.024^a^	2.007^a^
	7	1.102^a^	0.931^a^	1.066^a^	1.056^a^	1.457^a^	1.437^a^
	8	1.032^a^	0.865^a^	1.004^a^	1.002^a^	1.432^a^	1.400^a^
	9	0.654^a^	0.587^a^	0.687^a^	0.694^a^	0.935^a^	0.906^a^
	10	0.383^a^	0.423^a^	0.519	0.541^a^	0.758	0.723^a^
	11	–0.201^a^	–0.218^a^	–0.11	–0.075	–0.108	–0.139
	12	–0.392^a^	–0.517^a^	–0.333	–0.33	–0.371	–0.393
	13	–0.312^a^	–0.498^a^	–0.306	–0.29	–0.396	–0.407
	14	–0.393^a^	–0.484^a^	–0.333	–0.307	–0.437^a^	–0.44
	15	–0.654^a^	–0.722^a^	–0.556^a^	–0.530^a^	–0.647^a^	–0.659^a^
	16	–0.884^a^	–1.038^a^	–0.847^a^	–0.829^a^	–0.976^a^	–1.005^a^
	17	–0.984^a^	–1.255^a^	–1.061^a^	–1.011^a^	–1.188^a^	–1.193^a^
	18	–1.182^a^	–1.532^a^	–1.339^a^	–1.296^a^	–1.386^a^	–1.367^a^
	19	–1.491^a^	–1.913^a^	–1.673^a^	–1.613^a^	–1.684^a^	–1.649^a^
	20	–1.551^a^	–2.017^a^	–1.808^a^	–1.766^a^	–1.845^a^	–1.814^a^
	21	–1.759^a^	–2.240^a^	–2.025^a^	–2.000^a^	–2.014^a^	–1.978^a^
	22	–2.096^a^	–2.584^a^	–2.318^a^	–2.284^a^	–2.344^a^	–2.313 ^a^
	23	–2.106^a^	–2.550^a^	–2.280^a^	–2.250^a^	–2.263^a^	–2.251^a^
	24	–2.253^a^	–2.653^a^	–2.392^a^	–2.377^a^	–2.359^a^	–2.360^a^
	25	–2.300^a^	–2.629^a^	–2.427^a^	–2.409^a^	–2.489^a^	–2.497^a^
	26	–2.300^a^	–2.564^a^	–2.312^a^	–2.302^a^	–2.386^a^	–2.410^a^
	27	–2.371^a^	–2.550^a^	–2.281^a^	–2.292^a^	–2.268^a^	–2.306^a^
	28	–2.394^a^	–2.527^a^	–2.281^a^	–2.313^a^	–2.271^a^	–2.303^a^
	29	–2.418^a^	–2.465^a^	–2.229^a^	–2.270^a^	–2.190^a^	–2.200^a^
	30	–2.441^a^	–2.413^a^	–2.179^a^	–2.233^a^	–2.166^a^	–2.212^a^
	31	–2.488^a^	–2.446^a^	–2.162^a^	–2.256^a^	–2.173^a^	–2.203^a^
	32	–2.488^a^	–2.347^a^	–2.080^a^	–2.182^a^	–2.167^a^	–2.222^a^
	33	–2.512^a^	–2.299^a^	–2.013^a^	–2.142^a^	–2.112^a^	–2.187^a^

^a^ *P*<.05.

**Table 9:** Coefficient of determination (*R* ^2^) change and coefficient results for dependent variable flu symptom(s).

Independent variables		Model 2		Model 3		Model 4		Model 5		Model 6	
		Coefficent	*P*	Coefficent	*P*	Coefficent	*P*	Coefficent	*P*	Coefficent	*P*
**Flu and media**											
	Flu incidence	0.644	<.001	0.528	<.001	0.498	<.001	0.371	<.001	0.473	<.001
	Media			0.293	<.001	0.241	<.001	0.334	<.001	0.323	<.001
	Flu incidence × media					0.194	<.001	0.177	<.001	0.181	<.001
**Private resources**											
	Life quality							–0.401	.001	–0.389	.004
	Age							–0.143	.06	–0.208	.05
	Chronic disease							–0.279	.004	–0.309	.002
	Health lifestyles							–0.287	.009	–0.205	.02
**Public resources**											
	Public funds							–0.315	.05	–0.375	.04
	Population density							0.188	.23	0.169	.14
	Primary physicians							0.123	.06	0.106	.07
	Outpatient visits							1.455	<.001	1.523	<.001
	Hospital beds							–3.356	<.001	–3.601	<.001
	Hospital care utilization							0.601	.008	0.651	.02
**Interactions**											
	Flu × life quality									0.001	.03
	Flu incidence × age									0.173	.08
	Flu incidence × chronic disease									0.109	.09
	Flu incidence× healthy lifestyles									0.072	.30
	Flu incidence × public funds									–0.018	.88
	Flu incidence × population density									–0.095	.37
	Flu incidence × primary physician									0.150	.03
	Flu incidence × outpatient visits									–0.666	.52
	Flu incidence × hospital beds									0.708	.08
	Flu incidence × hospital care utilization									–0.115	.43

**Table 10:** Coefficient of determination (*R* ^*2*^) change and coefficient results for dependent variable flu shot(s).

Independent variables		Model 2		Model 3		Model 4		Model 5		Model 6	
		Coefficent	*P*	Coefficent	*P*	Coefficent	*P*	Coefficent	*P*	Coefficent	*P*
**Flu and media**											
	Flu incidence	0.477	<.001	0.414	<.001	0.383	<.001	0.251	<.001	0.173	.04
	Media			0.162	<.001	0.144	<.001	0.189	<.001	0.206	<.001
	Flu incidence × media					0.089	.001	0.112	<.001	0.128	.001
**Private resources**											
	Life quality							–0.070	.43	–0.018	.32
	Age							–0.013	.27	–0.008	.29
	Chronic disease							–0.083	.41	–0.036	.51
	Health lifestyles							0.114	.62	0.078	.73
**Public resources**											
	Public funds							–0.413	.02	–0.544	.02
	Population density							0.191	.001	0.117	.05
	Primary physicians							1.439	.006	1.561	.01
	Outpatient visits							0.022	.08	–0.234	.13
	Hospital beds							–0.662	.64	–0.918	.72
	Hospital care utilization							–0.05	.19	0.073	.05
**Interactions**											
	Flu × life quality									–0.223	.03
	Flu incidence × age									0.045	.08
	Flu incidence × chronic disease									0.018	.54
	Flu incidence× healthy lifestyles									0.018	.43
	Flu incidence × public funds									–0.165	.09
	Flu incidence × population density									0.073	.06
	Flu incidence × primary physicians									0.823	.47
	Flu incidence × outpatient visits										0.011	.2
	Flu incidence × hospital beds									0.808	.06
	Flu incidence × hospital care utilization									–0.281	.43

## Discussions

### Principal Results

Research on the correlation between the incidence of certain diseases and online information searches related to those diseases has increased in recent years. However, there has been little research on the effects of social factors on online information searches related to disease. In this paper, we demonstrate the usefulness of online search for queries related to a health risk as a measure of public health risk perception. We use publicly available data to demonstrate how such data can be used to provide insights into the factors that influence the public’s perception of health risks.

The results of our regression analyses provide strong support for our hypotheses: Web search volumes for flu-related queries as a measure of public health risk perception is predicted by the incidence of the flu and social factors, including media attention, and private and public resources. In addition to the independent impact of these social variables, we anticipated that the effect of incidence of the flu on public risk perception would be heightened by factors in the social environment. However, our models that incorporated the interaction effects between flu incidence and social factors did not add much to the explanatory power of our regression models. The social environment affects public health risk perception regardless of the incidence of diseases.

We modeled information searches for both risk prevention (flu shots) and risk diagnosis (flu symptoms). In our analyses, independent variables, especially media attention and private and public resources, had significant influence on search volumes for flu symptoms; however, seasonality variables had significant influence on search volumes for flu shots. As we anticipated, different factors appear to influence public perception of risk diagnosis and risk prevention.

Both the model for flu symptoms and that for flu shots demonstrate positive main effects for the incidence of the flu on search volumes. When a population’s flu incidence is higher, the population’s concerns for both prevention and diagnosis of the risk are higher.

Our data also support the expected positive effects of media attention and its interaction with the incidence of the flu on search volumes for risk prevention and risk diagnosis. Because mass media pays more attention to the risks related to a specific population, the overall population and the population with the flu both have more concern for prevention and diagnosis of the risk. Thus, our results show that the media play a significant role in setting the public agenda for health risk (agenda-setting theory [32]).

Private resources represented by life quality and public resources represented by hospital beds were negatively related to search volumes for flu symptoms. For the risk of the flu, a population with higher life quality and more access to hospital services demonstrated less searches for symptoms, whereas a population with lower life quality and less access to hospital services demonstrated more searches for symptoms. These results suggest consumers may use information from the Internet as a substitute for health care resources. Specifically, consumers vulnerable because of lower life quality and less available hospital services engage in more Internet searches, perhaps because information available on the Internet represents a relatively low-cost and easy-access source for information related to health risks.

With regard to private and public resources related specifically to health, our analyses suggest that there may be some synergistic effects of the 2 types of resources. Private resources represented by healthy lifestyles and public resources represented by outpatient visits and hospital care utilization are positively related to search volume for flu symptoms. Further, public resources represented by primary physicians and public funds were positively related to search volume for flu shots.

Based on these findings, we suggest that when a population has healthier lifestyles and more contact with health care professionals (through outpatient visits, emergency department visits, inpatient stays, availability of primary care physicians, and dedicated public health funds), it may be more conscious about current health risks. These results raise a question about the direction of the relationship between access to health care professionals and consumers’ searches for health information on the Internet. It could be that access to health care professionals stimulates consumers to be more vigilant about risk protective behaviors. If this is the case, primary care physicians and public health agencies play an important role in educating the public to take protective actions.

Private resources represented by chronic diseases had a negative effect on search volume for flu symptoms. For the risk of the flu, a population with higher incidences of chronic diseases demonstrated less searches for flu symptoms. This finding may reflect an environmental constraint on Internet access rather than a lack of interest in such information. As noted in a report from the Pew Research Center [62], adults living with chronic disease are significantly less likely than healthy adults to have access to the Internet. Individuals with chronic diseases are more likely to have regular contact with health professionals, also highlighting the important role health care providers play in patient education about health risks.

Finally, in our analyses, public resources represented by population density had a positive effect on search volume for flu shots. This finding is important in that, as a contagious illness, the search patterns for flu may also emerge for other communicable diseases.

### Implications

Our study has important implications for public policy makers and health care professionals theoretically and practically. First, based on ecological systems theory, we proposed that there is a correlation between online health information search and public health risk perception. Recognition of this relationship by policy makers and health care professionals is important. In designing health risk communications strategies and policies, it is critical to take the social environments in which the public engages in online health information search into consideration.

Second, we suggest that the analysis of Internet search query data related to a particular health risk can provide a bell-weather of public health risk perception. Our analysis suggests that online health information search is a reflection of public health risk perception that can be predicted by social context variables. We demonstrate a practical way for policy makers and health professionals to monitor these contextual factors. Previous work has shown that aggregate search data reflect public concerns or interests (eg, [63]). Following this stream of research, we demonstrate that when a population is concerned about a specific health risk, they engage in online searches about that risk. This online search is predicted by contextual factors. Monitoring these contextual variables on a regular basis can assist policy makers in identifying areas and/or populations that could benefit from enhanced education. It may also help identify areas on which to focus the development/expansion of resources.

The search volume data for queries representing different stages of risk response, such as prevention and diagnosis, can inform policy makers and health professionals about the likely response of a population to current and emerging threats. Social marketing resources should be allocated based on an understanding of public risk perception of prevention and diagnosis of a health risk. For example, seasonality had more influence on search volume related to prevention that did other variables. Social marketing efforts should be timed to coincide with the seasonal variation in public risk perception for flu prevention. Those states with high levels of public perception for risk prevention can use this finding to help prepare for a new flu season by arranging for extra supplies of flu vaccine and planning effective systems for distributing the vaccine. States with low levels of public risk perception for prevention might benefit from more health education and health promotion prior to the onset of a new flu season to help increase awareness of the impending risk.

We found that media attention and private and public resources had strong effects on public risk perception for symptoms. Populations with higher risk perception for the diagnosis of the flu are likely to have higher demand for products or services related to treatment of the flu (eg, vitamin C supplements, primary care visits). Retailers in states with high levels of public risk perception for response to the flu may want to ensure that they have adequate supply of over-the-counter medications for dealing with flu symptoms. Ambulatory care clinics and primary care providers can assist a population in dealing with the flu by providing educational materials focused on identification of symptoms and by ensuring same-day access to provider visits for patients experiencing flu symptoms. To respond to public risk perception for diagnosis of the flu, social marketing efforts should use sociocultural segmentation (eg, vulnerable and health conscious consumers) to target resources most needed by each segment.

With the popularity of mobile devices (eg, smartphones, iPads), mobile searches are growing among consumers. Surveys have shown that a search engine is the most used application by 77% of smartphone users, and 90% of mobile search activities result in actions (eg, purchasing, recommending) [64]. This suggests that search data from mobile devices may reflect the public’s perception of the urgency of the risks and their ability to manage the risks. We suggest that policy makers and health care professionals use mobile search data related to health risks to establish more actionable and timely strategies.

Online information searching is a bidirectional communication process, including sending search requests and receiving search results. Sending search requests reflects the public’s perception of the severity and urgency of risks, whereas receiving search results reflects the public’s perception of their ability to manage or respond to the risks. This study focused on public risk perception as demonstrated by the patterns of search requests. Policy makers and health professionals may further explore public risk perception by examining the patterns of responses to the returns to search requests. We suggest that the public’s perception of the management of health risks may be revealed through behaviors that reflect 4 types of social relations (ie, hierarchical, egalitarian, individualist, and fatalist social relations) [29]. People with a hierarchical approach to social relations (ie, supporting patriotism, law, and order) may be more likely to click on search results links from official government websites, whereas people with an individualist way of life (ie, supporting individual efforts) may be more likely to click on search results from citizen media (eg, independent journalists). We suggest that investigating the association between the public’s response to search results and social-cultural factors may be a practical way to assess the public’s perception of the management of health risks. Policy makers and health care professionals may combine the patterns of search requests and response to search results to generate a composite index for public health risk perception.

### Limitations

Our study has several limitations. First, data gaps exist for the variables we used to indicate flu incidence and online information searches related to the flu. By using a different unit of analysis, additional relevant data may be available for study. For example, for each flu season from 1997 to the current year, the CDC posts the data of ILI outpatient visit rates for 9 flu surveillance regions on its official websites [65]. Regional data are available for Pacific, Mountain, West South Central, East South Central, West North Central, East North Central, New England, mid-Atlantic, and South Atlantic. Using these data regional variations regarding public health risk perception could be explored. Similarly, with data that are available at the state level, state data could be combined, facilitating regional analysis. Such regional analyses may be particularly relevant to public health risks that occur most commonly in particular geographic areas, such as those related to hurricanes.

Second, we base our findings on aggregate data. One limitation of aggregate data is that they represent the characteristics of a group as a whole but do not allow for analysis of individual variation. We cannot establish how individuals perceive their social environments related to health risks. Future research is needed to investigate individual responses to social factors related to health risks by collecting data from self-report surveys.

Next, our study has only shown the usefulness of sets of variables for the prediction of public risk perception related to the flu. Different types of health risks vary in their characteristics such as immediacy, frequency, and severity. These factors may lead to variations not only in the effects of disease incidence, but also the relationship of sociocultural factors to public risk perception as demonstrated by online information search. More research is needed to identify common and unique variables for the measurement of public risk perception related to different types of health risks. For example, food-borne illnesses and the flu are both common health risks. Vaccination is available for the flu but not for food-borne illnesses. Future research should consider the availability of preventive and treatment options for different health risks as they may affect public perception for the health risks.

Finally, our study has shown the strong effects of traditional mainstream mass media (ie, newspaper and news wires) on public risk perception. Research is needed to investigate the influence of multiple forms of mass media, especially social media (eg, blogs, online social networks), on public risk perception. May’s [66] report about information channels and networks during Hurricane Katrina has identified the prominence of digital communication for risk management.

## Multimedia Appendix 1

Google Insights for Search (GIFS) Methodology.

## Abbreviations

GIFS: Google Insights for Search
ILI: influenza-like illness
