# Supplementary material for: Incidence of Online Health Information Search: A Useful Proxy for Public Health Risk Perception
Source: J Med Internet Res. 2013 Jun 17;15(6):e114. doi: 10.2196/jmir.2401 (PMC3713924; doi:10.2196/jmir.2401)
Supplement: Supplementary file 1 [file jmir_v15i6e114_app1.pdf]

Multimedia Appendix 1

Google Insights for Search (GIFS) Methodology

GIFS provides weekly or monthly relative web search volume for specific search queries within a specific time frame and for a specific geographic region (data available since January 2004). By entering the selected search queries in the entry boxes (one box for one query), and setting the region as a specific state and the time frame as the time period from 2004 to 2011, we can get weekly relative search volume data for specific queries.

GIFS provides several types of matching functions; we selected the matching function that shows results for searches containing both the plural and singular form of queries. Thus our three queries are: "flu", "flu symptom + flu symptoms", and "flu shot + flu shots" (e.g., "flu symptom + flu symptoms").

Normalization of Data by Google Insights for Search

Google Insights for Search (GIFS) allows one to enter up to five regions (e.g., state) for one query. For each entry, GIFS provides relative search volume data, which is normalized on a scale from 0-100. GIFS normalizes search volume data from one region by two steps. First, GIFS normalizes the search volume data by the total traffic during the set time frame for this region. For example, if we set the time frame as the year of 2004 and the state as Arizona, the search volume for one query for one week in 2004 will be reported as the number relative to the total search volume for this query from Arizona for the year of 2004. Then, GIFS normalizes search volumes by the region with the highest search volume. For example, for the comparison of the search volumes for a query from two states: Arizona and California, GIFS may report that the search volume for a specific week from Arizona reached the peak value of 100. The search volumes from California will be reported as the numbers relative to this peak value. For each query, we used search volumes for the state that has the highest search volumes as the benchmark. Thus, the search volumes for each state are reported

relative to the search volumes for the benchmark state.

For the two queries “flu symptom + flu symptoms”, and “flu shot + flu shots”, the benchmark states with an index of 100 in peak weeks is respectively, Arizona and Rhode Island. Other states are indexed to these benchmark states. The data are not available for all the states for each query. In all, the dataset includes search volume data for 25 states for the query “flu symptom + flu symptoms”, and 39 states for the query “flu shot + flu shots” over a time period from 2004 to 2011.
